# Phylogenomic analysis of *Balantidium ctenopharyngodoni* (Ciliophora, Litostomatea) based on single-cell transcriptome sequencing

**DOI:** 10.1051/parasite/2017043

**Published:** 2017-11-14

**Authors:** Zongyi Sun, Chuanqi Jiang, Jinmei Feng, Wentao Yang, Ming Li, Wei Miao

**Affiliations:** 1 Hubei Key Laboratory of Animal Nutrition and Feed Science, Wuhan Polytechnic University, Wuhan 430023 PR China; 2 Institute of Hydrobiology, Chinese Academy of Sciences, No. 7 Donghu South Road, Wuchang District, Wuhan 430072, Hubei Province PR China; 3 Department of Pathogenic Biology, School of Medicine, Jianghan University, Wuhan 430056 PR China

**Keywords:** *Balantidium ctenopharyngodoni*, Trichostomatia, single-cell transcriptome sequencing, SSU rDNA, phylogenomic analysis

## Abstract

In this paper, we present transcriptome data for *Balantidium ctenopharyngodoni* Chen, 1955 collected from the hindgut of grass carp (*Ctenopharyngodon idella*). We evaluated sequence quality and *de novo* assembled a preliminary transcriptome, including 43.3 megabits and 119,141 transcripts. Then we obtained a final transcriptome, including 17.7 megabits and 35,560 transcripts, by removing contaminative and redundant sequences. Phylogenomic analysis based on a supermatrix with 132 genes comprising 53,873 amino acid residues and phylogenetic analysis based on SSU rDNA of 27 species were carried out herein to reveal the evolutionary relationships among six ciliate groups: Colpodea, Oligohymenophorea, Litostomatea, Spirotrichea, Heterotrichea and Protocruziida. The topologies of both phylogenomic and phylogenetic trees are discussed in this paper. In addition, our results suggest that single-cell sequencing is a sound method of obtaining sufficient omics data for phylogenomic analysis, which is a good choice for uncultivable ciliates. The transcriptome data for *Balantidium ctenopharyngodoni* are the first omics data within the subclass Trichostomatia, and provide a good basis for ciliate phylogenomic analysis, as well as related omics analysis.

## Abstract (fr)

Les données du transcriptome de *Balantidium ctenopharyngodoni* Chen, 1955, recueillies à partir de l'intestin de la carpe herbivore (*Ctenopharyngodon idella*), sont présentées dans cet article. Nous avons évalué la qualité de sa séquence et avons assemblé de novo un transcriptome préliminaire, incluant 43,3 mégabits et 119 144 transcriptions. Ensuite, nous avons obtenu un transcriptome final, incluant 17,7 mégabits et 35 560 transcriptions, en supprimant les séquences contaminatrices et redondantes. Une analyse phylogénomique basée sur une supermatrice avec 132 gènes comprenant 53 873 résidus d'acides aminés et une analyse phylogénétique basée sur l'ADNr SSU de 27 espèces a été réalisée pour révéler les relations évolutives entre six groupes de ciliés: Colpodea, Oligohymenophorea, Litostomatea, Spirotrichea, Heterotrichea et Protocruziida. Les topologies des arbres phylogénomiques et phylogénétiques sont discutées dans ce document. En outre, nos résultats suggèrent que le séquençage d'une seule cellule est une méthode solide pour obtenir suffisamment de données omiques pour l'analyse phylogénomique, ce qui est un bon choix pour les ciliés incultivables. Les données du transcriptome de *Balantidium ctenopharyngodoni* sont les premières données omiques dans la sous-classe Trichostomatia et fournissent une base pour l'analyse phylogénomique des ciliés ainsi que l'analyse omique associée.

## Introduction

The genus *Balantidium* Claparède & Lachmann, 1858 belongs to the order Vestibuliferida (Trichostomatia, Ciliophora). It was established by Claparède and Lachmann when they separated *Bursaria entozoon* Ehrenberg, 1838 from the genus *Bursaria* Ehrenberg, 1838 and assigned it to *Balantidium entozoon* [4]. A large number of Balantidium species, thereafter, have been reported as endocommensals in the digestive tracts of both vertebrate and invertebrate hosts, especially fish and amphibians. At present, 19 fish-isolated species (13 freshwater species and 6 marine species) and 31 amphibian-isolated species (26 species from anura amphibians and 5 species from urodele amphibians) have been found [21].

*Balantidium ctenopharyngodoni* Chen, 1955 is a native species of China and a common obligatory intestinal parasite of grass carp (*Ctenopharyngodon idella*). It was first discovered and simply documented by Chen [2], then, its morphology redescribed [20], pathogenicity [28], ultrastructural anatomy [22] and related molecular phylogeny [22,24] were studied, respectively. As for phylogenetic analysis, the related studies reconstructed the phylogenetic trees only based on the small subunit ribosomal DNA (SSU rDNA) gene. The SSU rDNA gene is the most common gene marker for phylogenetic analysis [42], which was first used by Lynn et al. [23] to demonstrate divergences among ciliates over 25 years ago. For example, it was applied to place the hymenostomes and peritrichs as sister lineages [26,27]. However, with the development of high-throughput sequencing in recent decades, enormous amounts of omics data have become available for phylogenetic analysis, potentially including hundreds of thousands of loci, which provide much more information compared to single or multi-gene analysis [7,18,25]. To take full advantage of this abundant data, phylogenomics −research into evolutionary relationships based on comparative analysis of omics data − was proposed to replace single-gene phylogenetics [30,33], through the use of genomics rather than single-gene information [29]. This makes it possible to avoid problems related to sufficient sample size by addition of more sites from multiple genes or to compensate for biased base compositions. Although some of these problems will also affect phylogenies reconstructed from large datasets, others will be substantially reduced and/or easily diagnosed [5]. To date, several ciliates have been sequenced and analyzed, such as *Tetrahymena thermophila* [10], *Paramecium tetraurelia* [1], *Ichthyophthirius multifiliis* [6], *Pseudocohnilembus persalinus* [43] and *Oxytricha trifallax* [39], and the results obtained were used for phylogenomic analysis [3,11,12]. Along with the steady decline in costs as new technologies have been developed and/or refined, sequencing projects have provided a possible method, single-cell sequencing, for many uncultivable non-model organisms to fill in the gaps in phylogenomics, such as for *Balantidium*. Here we obtained RNA-Seq data for *B*. *ctenopharyngodoni*, which are the first omics data within the subclass Trichostomatia. Our present study also provided a good example of how to process the omics data from single-cell transcriptome sequencing.

## Materials and methods

### Specimen collection, isolation and identification

The grass carp (*C. idella*) used in this study were captured from Niushan Lake Fishery (30°19′N, 114°31′E) in Wuhan City, Hubei Province, China from August to October 2015 and transported alive to the laboratory for further examination. First, all fish samples were quickly dissected. Second, the intestines, especially hindgut, were opened to collect intestinal contents into petri dishes using tweezers and scissors. Third, 0.65% saline solution was added to the contents and we then waited for a few minutes to allow the ciliates to swim out of the intestinal contents. Finally, the ciliates were observed with a dissection microscope and collected by using dissecting needles and glass micropipettes.

Living specimens were observed, identified, and photographed using an OLYMPUS (BX51) camera. Some other specimens were also fixed in saturated HgCl_2_ solution and stained with protargol [36].

### Single-cell RNA-sequencing

The ciliates were isolated with a capillary under a dissection microscope and isolated cells were placed into 0.65% NaCl to starve more than 2 hours. Eight cells were picked from the starved cells and were then put into a 0.2-ml thin-walled PCR tube containing 2 µl of cell lysis buffer (0.2% (vol/vol) Triton X-100 and 2 U µl^−1^ RNase inhibitor) and kept in a volume as low as possible (preferably less than 0.5 µl). Then, the collected cells were frozen and stored at −80^⌊^ until further use (Figure S1). These cells were amplified by using Smart-Seq2 (SMARTer Ultra Low RNA Kit for Illumina sequencing; insert size about 350 bp) [31]. The sample of cDNA was sequenced for 150 bp at both ends (paired-end) by using an Illumina HiSeq4000 sequencer.

### Transcriptome assembly and analysis

The raw data of inferior quality reads were filtered by the fastq_quality_filter (-q 20, -p 80) in the FASTX-Toolkit (http://hannonlab.cshl.edu/fastx_toolkit/). Then, the transcriptome was *de novo* assembled using Trinity with the default parameter [13]. Two methods were used to filter the contaminants. During sample preparation, the ciliates were washed several times with 0.65% saline solution and sterile water (see above). During the bioinformatics analysis, firstly, transcripts were searched against the non-redundant nucleotide (nt) database from NCBI by using the Basic Local Alignment Search Tool (BLAST) (-E-value 0.01). Secondly, alignment results were divided into two parts: transcript fragments with hits and without hits (Figure S1). For the transcript fragments with hits, if the top hit belongs to Alveolata, the transcripts were reserved for further analysis; otherwise they were excluded from the transcriptome. Then, both the transcript fragments with hits belonging to Alveolata and transcript fragments without hits were put together and removed the redundancy of remnant transcriptome (transcripts of no-hits and top hit matching Alveolata) by using Corset [8] with default parameters. Finally, we obtained a contamination-exclusion and non-redundant transcriptome.

We used the contamination-exclusion and non-redundant transcriptome to carry out further analysis. Potential rDNA sequences were extracted from the transcripts, and species identification were verified by BLAST searching against the GenBank database by using the rDNA sequences as queries. Predicted protein sequences were obtained by translating the assembled transcripts using the ciliates' codon table (only TGA as stop codon, such as Tetrahymena) by the getorf program of the EMBOSS site [32] (table 6) and we picked the longest protein sequences from every translated sequence. Subsequently, we used interproscan-5.19-58.0 [16] with default parameters to implement functional annotation about these protein sequences and then classified the annotated result with WEGO (http://wego.genomics.org.cn/cgi-bin/wego/index.pl) [44]. For phylogenomic analysis, translated protein sequences, which are more than 200 amino acid residues, were selected to find orthologous genes.

### Data sources

Omics data and SSU rDNA gene sequences used in this study are listed in Table S1. Genomic data for four *Tetrahymena* spp. (*T. borealis*, *T. elliotti*, *T. malaccensis* and *T. thermophila*) were downloaded from the Tetrahymena Genome Database (TGD) (http://ich.ciliate.org/index.php/home/welcome); *Ichthyophthirius multifiliis*, *Oxytricha trifallax*, *Paramecium tetraurelia* and *Stylonychia lemnae* were obtained from the *Ichthyophthirius multifiliis* Genome Database (IchDB) (http://ich.ciliate.org/index.php/home/welcome/) the *Oxytricha* Genome Database (OxyDB) (http://oxy.ciliate.org/index.php/home/welcome), the ParameciumDB (http://paramecium.cgm.cnrs-gif.fr/) and the *Stylonychia* Genome Database (StyloDB) (http://stylo.ciliate.org/index.php/home/welcome/), respectively. Genomic data for two *Plasmodium* spp. (*P. falciparum* and *P. yoelii*) were downloaded from the PlasmoDB (http://plasmodb.org/plasmo/). Omics data for *Pseudocohnilembus persalinus*, *Paralembus digitiformis*, *Colpoda aspera*, *Campanella umbellaria,* and *Carchesium polypinum* were downloaded from the National Center for Biotechnology Information (NCBI) with accession numbers SRR1768438, SRR1768439, SRR1768440, SRR1768423, and SRR1768437, respectively. All transcriptomic data of the eleven ciliate species were downloaded from iMicrobe (http://imicrobe.us/) under project numbers MMETSP0125: *Aristerostoma* sp., MMETSP0210: *Condylostoma magnum*, MMETSP0205: *Euplotes focardii*, MMETSP0213: *Euplotes harpa*, MMETSP0209: *Litonotus* sp., MMETSP0127: *Platyophrya macrostoma*, MMETSP0216: *Protocruzia adherens*, MMETSP0211: *Pseudokeronopsis riccii*, MMETSP0123: *Schmidingerella arcuata*, MMETSP0126: *Strombidinopsis acuminatum*, and MMETSP0208: *Strombidium inclinatum*. We obtained orthologous genes between two species by using the Reciprocal Best Hits (RBH) approach in BLAST with E-value no more than 10^−20^. If the score of the second-best hit of either gene in the other genome was less than half of the score of the best hit, the RBH-pair was retained. Only gene sets including more than 70% (at least 19 different species of our 27 species) of all species were retained for further analysis [11].

The SSU rDNA gene sequence for *B. ctenopharyngodoni* was obtained from an assembled 1950-bp fragment (Figure S2) in this work. The SSU rDNA gene sequences of the other 27 species were downloaded from GenBank and iMicrobe.

### Sequence alignment and phylogenetic analysis

For phylogenomic analysis, sequence alignments were implemented by MUSCLE version 3.6 [9], with the default parameters. Sequence alignments were re-optimized using Gblock 0.91b (−t = d, −b2 = 0.65, −b3 = 10, −b4 = 5, −b5 = a) to detect and trim the ambiguously aligned regions [40]. The 132 individual alignments of the 27 taxa were concatenated into a supermatrix by SCaFoS software version 4.42 using the data set assembling panel [35]. The maximum-likelihood (ML) analysis and Bayesian inference (BI) analysis followed Feng et al. [11]. For ML analysis, the concatenated data set was conducted in RAxML version 7.2.6 [38], with the LG model of amino acid substitution + F + Γ4 distribution (Γ four rate categories). The ML analysis was evaluated with 100 replicates. For BI analysis, the concatenated data set was implemented by the software PhyloBayes 3.3 [17] under CAT-POI model + Γ4 (a discrete gamma distribution of rate variation with four rate categories) with two independent Markov chain Monte Carlo (MCMC) runs. In order to evaluate the convergence of the two independent MCMC runs, the bpcomp program was used to compare the discrepancy of bipartition frequencies between the two runs and output a consensus tree and the maxdiff equalled 0.068, which was less than 0.1.

For the SSU rDNA gene, sequence alignments were implemented by ClustalW algorithm in MEGA6, with the default parameters (Gap Extension Penalty: 6.66; Transition Weight: 0.5) [41]. In order to decrease the number of characters presenting ambiguous alignment, we trimmed the ambiguously aligned positions. Our final data set comprised 1,695 nucleotide sites, which were used for subsequent analysis. A phylogenetic tree based on the SSU rDNA gene was constructed by the maximum-likelihood (ML) method with the MEGA6 and Bayesian Inference (BI) method with MrBayes 3.2 [34], respectively. For the ML analysis, the Tamura 3-parameter model of nucleotide substitution with a proportion of invariable sites (I) and a Gamma distribution (G) (T92 + G + I) as the best model of nucleotide evolution based on the Akaike Information Criterion [15] were used to calculate in MEGA6. For the BI analysis, a GTR + G + I model (n = 6, rates = invgamma) was selected to perform for 1,000,000 generations (or until the standard deviations of split frequencies were below 0.01) with trees sampled every 1,000 generations and an initial relative burn-in of 25% of the trees by MrBayes 3.2 and other parameters were default.

## Results

### Morphological description

**Figure 1:**
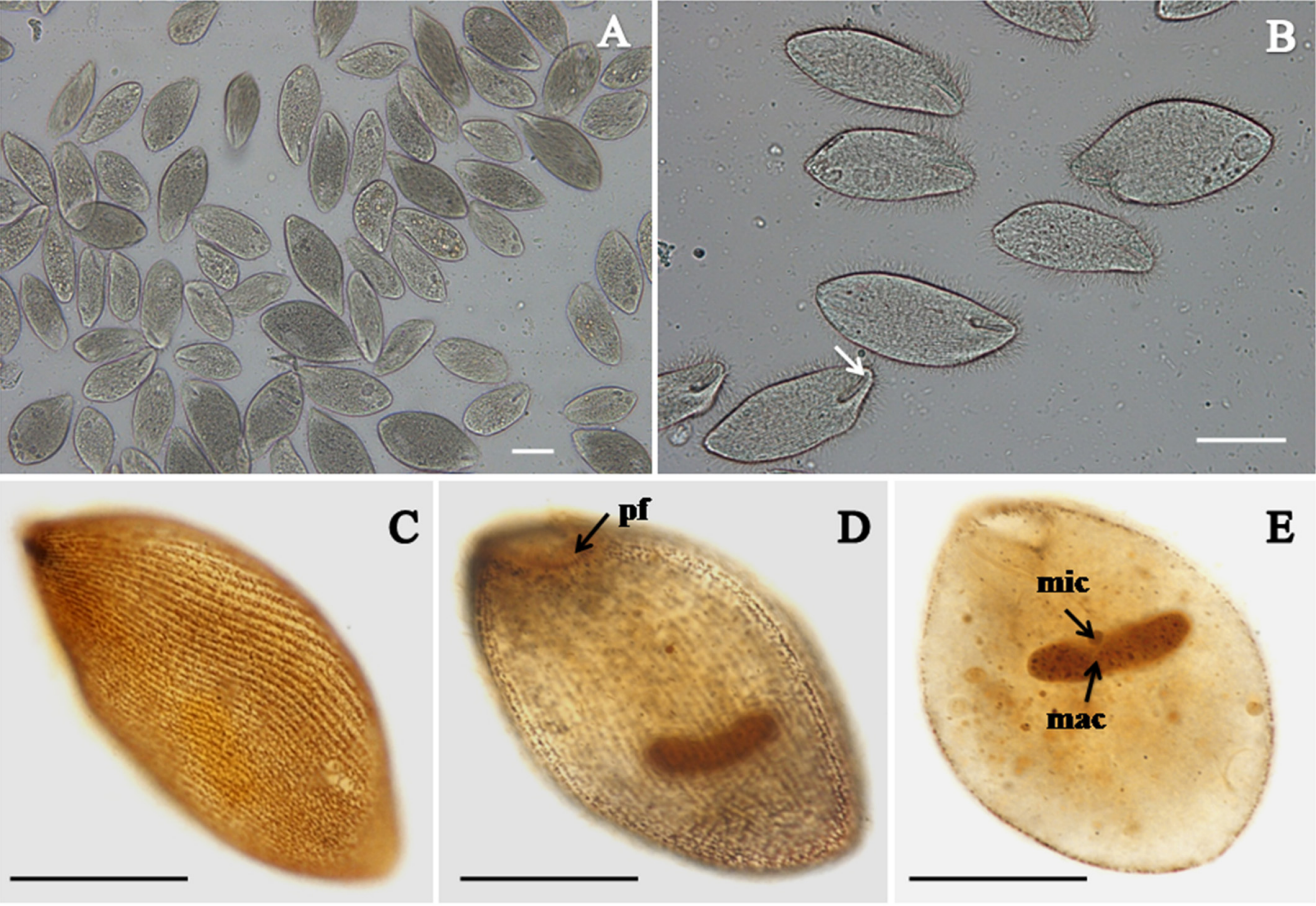
Light microscopy images of *Balantidium ctenopharyngodoni*. A. Living specimens, showing numerous *B. ctenopharyngodoni* specimens in the luminal contents of the hindgut. Scale bar = 50 µm. B. Living specimens, showing the general form and vestibulum (arrow). Scale bar = 50 µm. C-D-E. Specimens stained with protargol, showing its somatic kineties (C.) peripheral fibres (pf), (D.) macronucleus(mac) and micronucleus (mic), (E.). Scale bar = 50 µm.

*B. ctenopharyngodoni* specimens are mainly found in the luminal contents of the hindgut, especially near the anal opening. The organism is spindle-like or oval in shape (Figure 1A). Body size 76.2 − 160.7 µm ( = 98.0 µm; n = 30) × 40.5 − 70.1 µm ( = 59.2 µm; n = 30). Its body is highly flexible to permit the ciliate to move through narrow intestinal folds. The vestibulum is situated anteroventrally, extending directly posterior to about one sixth of the body length (Figure 1B). All ciliary rows started from the border of the vestibulum, arranged in closely set lines parallel to the longitudinal axis and packed over the body (Figures 1C and D). The macronucleus is kidney-shaped, usually with a tiny spherical micronucleus embedded in the middle concavity (Figure 1E).

## Single-cell RNA-sequencing and transcriptome analysis

The Illumina HiSeq4000 sequencer produced a total of 19,440,851 paired-end reads (averaged 150 bp for each read) for *B. ctenopharyngodoni* (including 5,832,255,300 bases with 34% average GC content), and 96.88% reads were retained after filtering low quality reads. The remaining high-quality reads were used to assemble the transcriptome and a 43.3 megabit (Mb) preliminary redundant transcriptome was obtained, including 119,141 transcripts. To exclude contamination, 119,141 transcripts were searched against nt database by using BLAST. In the result, 40,411 transcripts were hits and 78,730 transcripts had no hits. Moreover, the top hit of 40,236 transcripts belonged to non-Alveolata and the top hit of 175 transcripts belonged to Alveolata among the 40,411 transcripts. As a result, we discarded 40,236 transcripts from the preliminary redundant transcriptome to gain redundant transcriptome, including 78,905 transcripts. To remove redundancy from the new transcriptome, 43,345 transcripts were abandoned by using Corset with default. In the end, we obtained a final transcriptome including 35,560 transcripts (Figure S1). We implemented functional annotation of 35,560 transcripts with WEGO, and 1,427 transcripts were annotated and classified (Figure S3).

Two fragments, the 698 bp fragment and the 1277 bp fragment, of SSU rDNA sequences were extracted from the transcriptome assembled here by using two rDNA sequences (GU480804 and KU170972) of *B. ctenopharyngodoni* from GenBank as queries. Sequence alignments (Figure S2) showed that there is a 25-bp overlap between the two fragments, which leads to a 1950 bp potential rDNA. The 1950 bp rDNA sequence shows 99.69% identity to the GU480804 with the alignment region from 19 to 1368 bp. The 1950 bp rDNA sequence shows 100% identity to the KU170972 with the alignment region from 1 to 330 bp.

### Phylogenomic analysis

**Figure 2:**
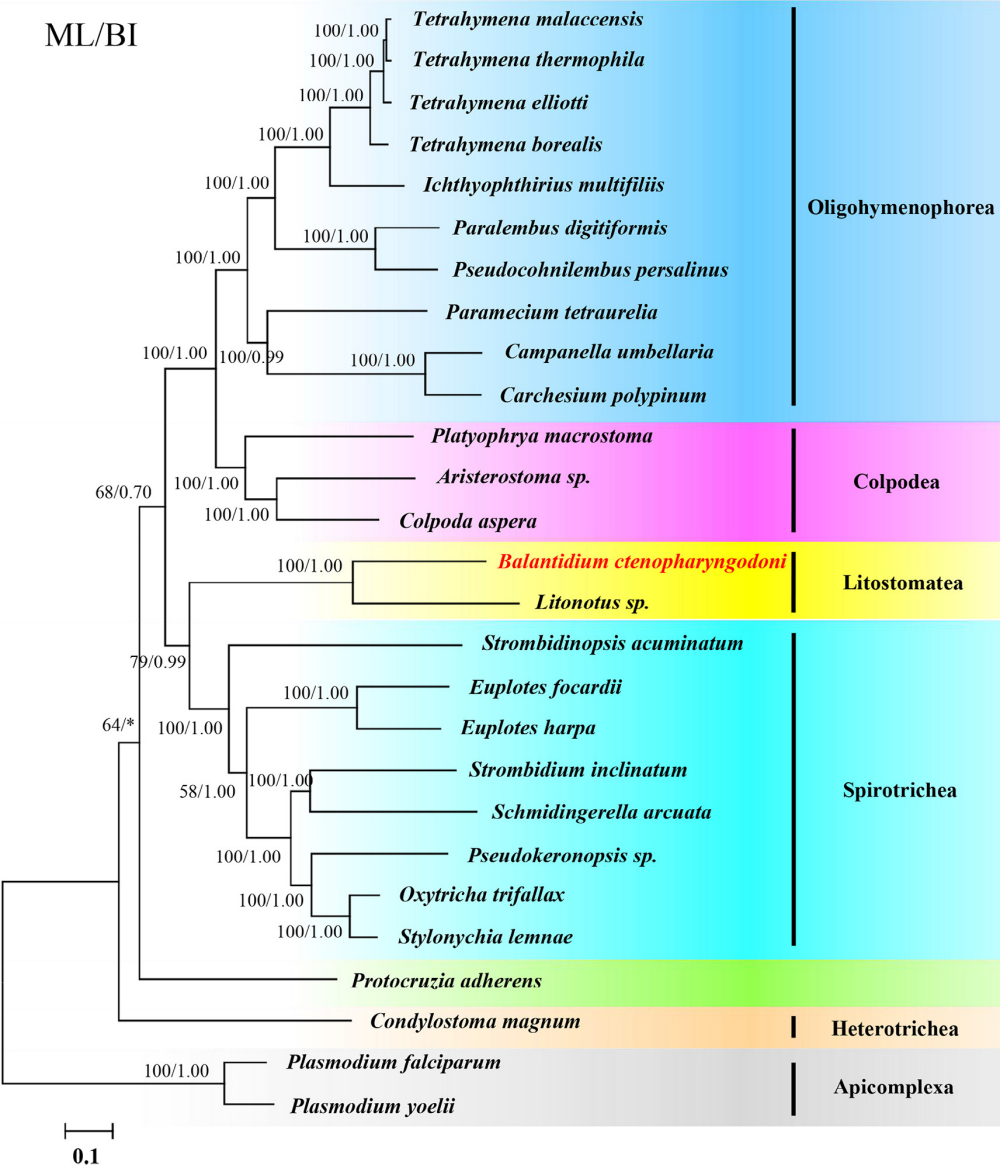
Phylogenomic relationships of ciliates. ML tree topology of a phylogenomic matrix comprising 53,873 unambiguously aligned amino acid residues of 27 taxa were inferred by using RAxML software under the LG + F + Γ4 model. Two *Plasmodium* spp. were used as outgroups. New sequencing species in this study are in red script with yellow shading. Numbers at nodes are ML bootstrap values followed by BI posterior probability. Bootstrap values from 100 replicates are given on the nodes. The scale bar corresponds to 0.1 expected substitutions per site.

We presented the taxonomic sampling of 27 species by integrating data from one newly obtained transcriptome (*B. ctenopharyngodoni*) and other omics data from NCBI, TGD, IchDB, OxyDB, ParameciumDB, StyleDB and iMicrobe. Altogether, omics data for 25 ciliates were used in our analysis and two species of *Plasmodium* were used as the outgroups. The 25 ciliate samples included six ciliate groups: Colpodea, Oligohymenophorea, Litostomatea, Spirotrichea, Heterotrichea and Protocruziida. We assembled a 132-gene dataset comprising 53,873 amino acid residues of the 27 species by analyzing their genome and transcriptome data. Both the ML tree and BI tree show a consistent topological tree. In addition, the ML tree topology is presented with support values of both ML and BI analysis indicated on branches (Figure 2). In the phylogenomic tree, Colpodea is sister to Oligohymenophorea and Litostomatea is sister to Spirotrichea. The phylogenomic analysis provides full statistical support that *B. ctenopharyngodoni* is sister to *Litonotus* sp., and both of them are Litostomatea. *Protocruzia adherens* and *Condylostoma magnum* are placed in the earliest diverging position in the ciliate tree.

**Figure 3:**
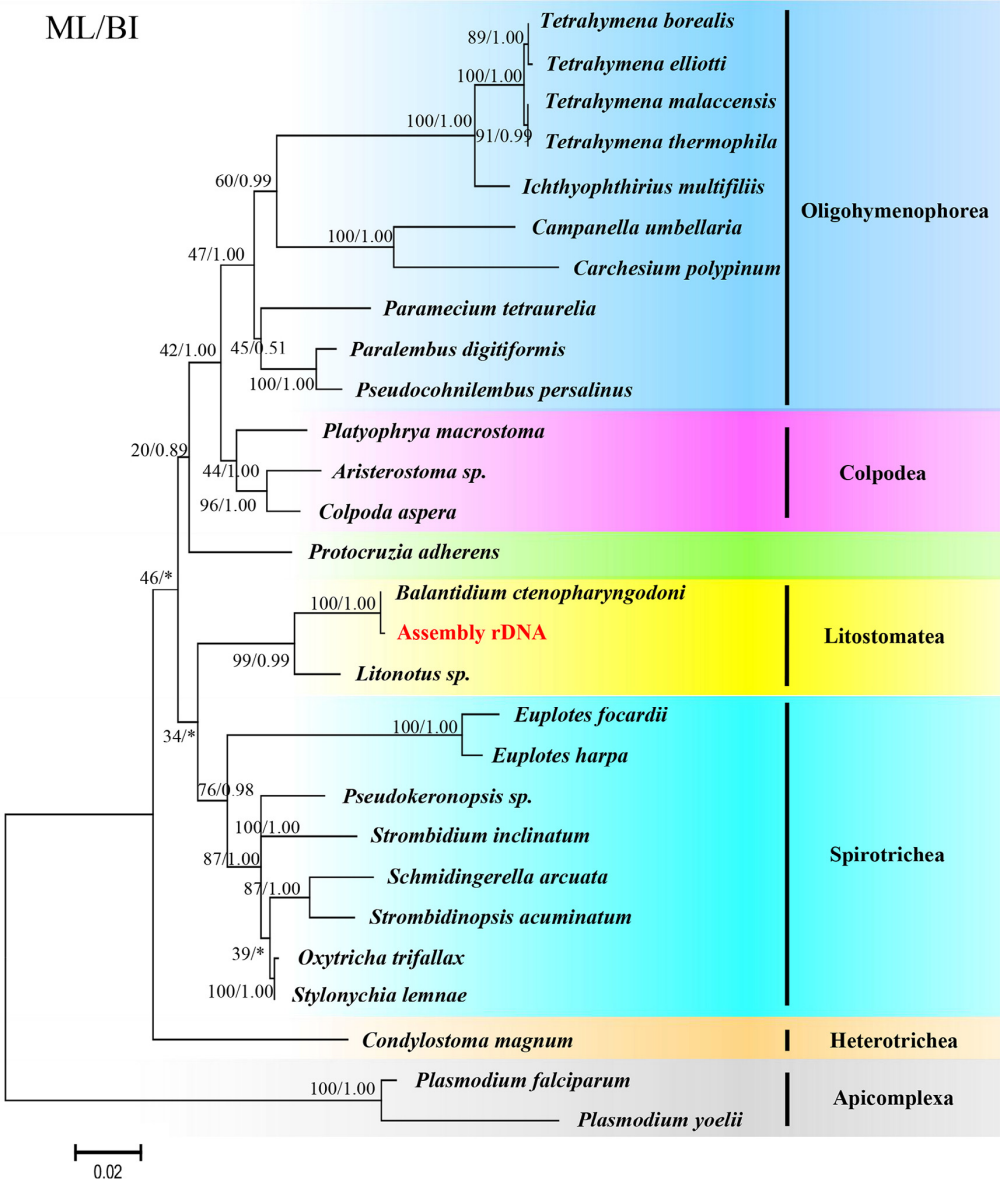
Phylogenetic tree based on the SSU rDNA sequences of ciliates. The ML tree was implemented by MEGA6 using the Tamura 3-parameter model, and Bayesian Inference by Mrbayes 3.2 with the GTR + G + I model. Two *Plasmodium* spp. were used as outgroups. The scale bar corresponds to 0.02 expected substitutions per site. The sequence extracted from the present transcriptome is in red script with yellow shading.

The topology of the phylogenetic tree derived from the SSU rDNA genes reconstructed by ML and BI is very similar to that of the phylogenomic tree. However, the phylogenetic tree derived from the SSU rDNA genes (Figure 3) showed that Peritrichia (*Campanella umbellaria* and *Carchesium polypinum*) is sister to the Hymenostomatia (*Tetrahymena* spp. and *Ichthyophthirius multifiliis*), and Peniculia (*Paramecium tetraurelia*) is sister to Scuticociliattia (*Pseudocohnilembus persalinus* and *Paralembus digitiformis*). Moreover, *Protocruzia adherens* is sister to Colpodea and Oligohymenophorea, but not in the deepest branch.

## Discussion

The important morphological characteristics of *B. ctenopharyngodoni* observed herein are consistent with those described by Li et al. [20]. Combined with evidence from the SSU rDNA sequences, we confirmed that the ciliate isolated in the present study is *B. ctenopharyngodoni*.

For single-cell sequencing, there is also a problem in transcriptome assembly of ciliates which are uncultivable, such as *B. ctenopharyngodoni*. When we collected the experimental sample, some contaminations were inevitably included. It was therefore very important concerning uncultivable ciliates to exclude contamination before implementing bioinformatics analysis. In this work, contaminants were excluded in two steps: (1) eradicating surface impurities of ciliates; (2) eliminating sequences of residual contaminants. In the first step, a simple and effective method is to clean ciliates by washing with distilled water. However, since many ciliates are sensitive to osmotic pressure, cleaning with distilled water may result in cell lysis. We found that *B. ctenopharyngodoni* remained alive for about 10 minutes in distilled water and most of them could be kept alive for more than 30 minutes in 0.65% NaCl, while others survived more than 2 hours. As a result, we starved *B. ctenopharyngodoni* for about 2 hours in 0.65% NaCl before collecting samples. This was done because keeping the microorganisms alive for a long time to digest the intracellular contamination (food residue) is a very effective method of reducing contamination. Washing several times with 0.65% saline and distilled water was done to remove epiphytic contamination. In the second step, excluding contamination by using bioinformatics analysis, we *de novo* assembled transcriptome directly and then searched against the nt database to discard the contaminating transcripts and redundant ones (see above). Furthermore, guanine-cytosine (GC)-content is a useful feature of ciliate sequences to distinguish them from one another [43]; thus, we used the GC-content distribution to evaluate the quality of the transcriptome assembled here. The GC-content distribution of the preliminary redundant transcriptome (Figure S4) of the sequencing sample *B. ctenopharyngodoni* shows two peaks, a main peak (32%) and a sub-peak (50%), indicating that some other sequences were present in the preliminary redundant transcriptome. After excluding contamination and reducing redundancy by bioinformatics analysis, there was only one peak (32%) in each of the two new transcriptomes, indicating that the method of bioinformatics analysis to exclude contamination was very efficient. Therefore, we suggest that integrated methods to reduce contamination using sterile isosmotic solution to wash ciliates is a good way to exclude contamination. Moreover, poly-A is a specific characteristic of eukaryote mRNA and is a target for ciliates to obtain transcripts before sequencing, which can in theory filter bacterial contamination. Sequencing transcriptome is therefore a relatively sound method for uncultivable ciliates to be applied in phylogenomic analysis based on single-cell sequencing.

Phylogenomic trees show similar topological structures to the tree topology derived from the SSU rDNA gene. However, there is some debate on the phylogenetic position of Oligohymenophorea and *Protocruzia adherens* by comparing with the tree from these two methods. For Oligohymenophorea, the phylogenetic tree derived from the SSU rDNA gene shows that Peritrichia (*Campanella umbellaria* and *Carchesium polypinum*) is close to Hymenostomatia (*Tetrahymena* spp. and *Ichthyophthirius multifiliis*). By contrast, the phylogenomic tree derived from the supermatrix of the 132 gene containing 53,873 unambiguously aligned amino acid residues of 27 taxa supports the hypothesis that Scuticociliattia (*Pseudocohnilembus persalinus* and *Paralembus digitiformis*) is closer to Hymenostomatia (*Tetrahymena* spp. and *Ichthyophthirius multifiliis*). The taxonomic relationships among Peritrichia, Scuticociliattia and Hymenostomatia in Oligohymenophorea were previously controversial [11], and phylogenomic analysis has revealed that Scuticociliattia is sister to Hymenostomatia, but not Peritrichia. Concerning *Protocruzia adherens*, the marine benthic ciliate genus *Protocruzia* of has a long history of ambiguous taxonomy. Some molecular studies have shown that *Protocruzia* could be more closely related to Spirotrichea [14,37]. Similarly, some researchers have proposed that *Protocruzia* be given its own lineage status, although they failed to define the taxon [19]. Recently, Gentekaki et al. [12] discussed the taxon *Protocruzia* in detail based on phylogenomic analysis and postulated that *Protocruzia* is not a spirotrich. This incongruence may be caused by insufficient data, long branch attraction artifact or species imbalance. High-throughput sequencing can provide plentiful nucleotide sequence information to be used for phylogenomic analysis, which can provide more representative genes to construct phylogenetic trees and is conducive for us to reveal the true evolutionary relationships among species. Therefore, phylogenomic analysis is superior to phylogenetic analysis derived from single genes to discuss taxonomic placement when sufficient omics data are available. As for the uncultivable species, the sample preparation for high-throughput sequencing is a great challenge. Fortunately, the rapidly developing single-cell sequencing technology has helped to resolve this problem.

No interesting gene families or metabolic pathways were found by Gene Ontology (GO) annotation, KEGG pathway analysis (data not shown) and searching against the non-redundant protein (nr) database (data not shown), which probably results from the fact that the present transcriptome is incomplete, and complete annotation for analyzing the function of genes is unavailable. Therefore, further research should aim to sequence the genome of *B. ctenopharyngodoni* and resequence its transcriptome.

Thus far, more than 8,000 ciliates have been discovered. Unfortunately, most of them are uncultivable, such as rumen ciliates, *Trichodina* spp., *Balantidium* spp. and so on, making it difficult to ascertain their taxonomy. Our present results suggest that the methods in this paper are a sound way of obtaining sufficient omics data for phylogenomics analysis to solve this problem.

## Supplementary materials

**Table S1** List of the omics data and SSU rDNA sequences used in the present study.
**Figure S1** The flowchart for obtaining transcriptome without contaminations and redundancy.
**Figure S2** Sequence alignments of SSU rDNA of *B. ctenopharyngodoni*. Black script, the length of fragment; blue script, the start site and termination site of alignment; red script, the identity of alignment; gray bar, two transcripts extracted from assembled transcriptome; green bar, GU48080 (*B. ctenopharyngodoni* small subunit ribosomal RNA gene, partial sequence); yellow bar, KU170972 (*B. ctenopharyngodoni* internal transcribed spacer 1, partial sequence; 5.8S ribosomal RNA gene, complete sequence; and internal transcribed spacer 2, partial sequence).
**Figure S3** Gene Ontology (GO) annotation of the final transcriptome.
**Figure S4** The guanine-cytosine (GC)-content distribution of *B. ctenopharyngodoni*'s transcriptome. Yellow line, the GC content of preliminary redundant transcriptome; green line, the GC content of redundant transcriptome; red line, the GC content of final transcriptome. The Supplementary Material is available at http://www.parasite-journal.org/10.1051/parasite/2017043/olm
