# Supplementary figures and images for: Phylogenomic analysis of Balantidium ctenopharyngodoni (Ciliophora, Litostomatea) based on single-cell transcriptome sequencing
Source: Parasite. 2017 Nov 14;24:43. doi: 10.1051/parasite/2017043 (PMC5684829; doi:10.1051/parasite/2017043)

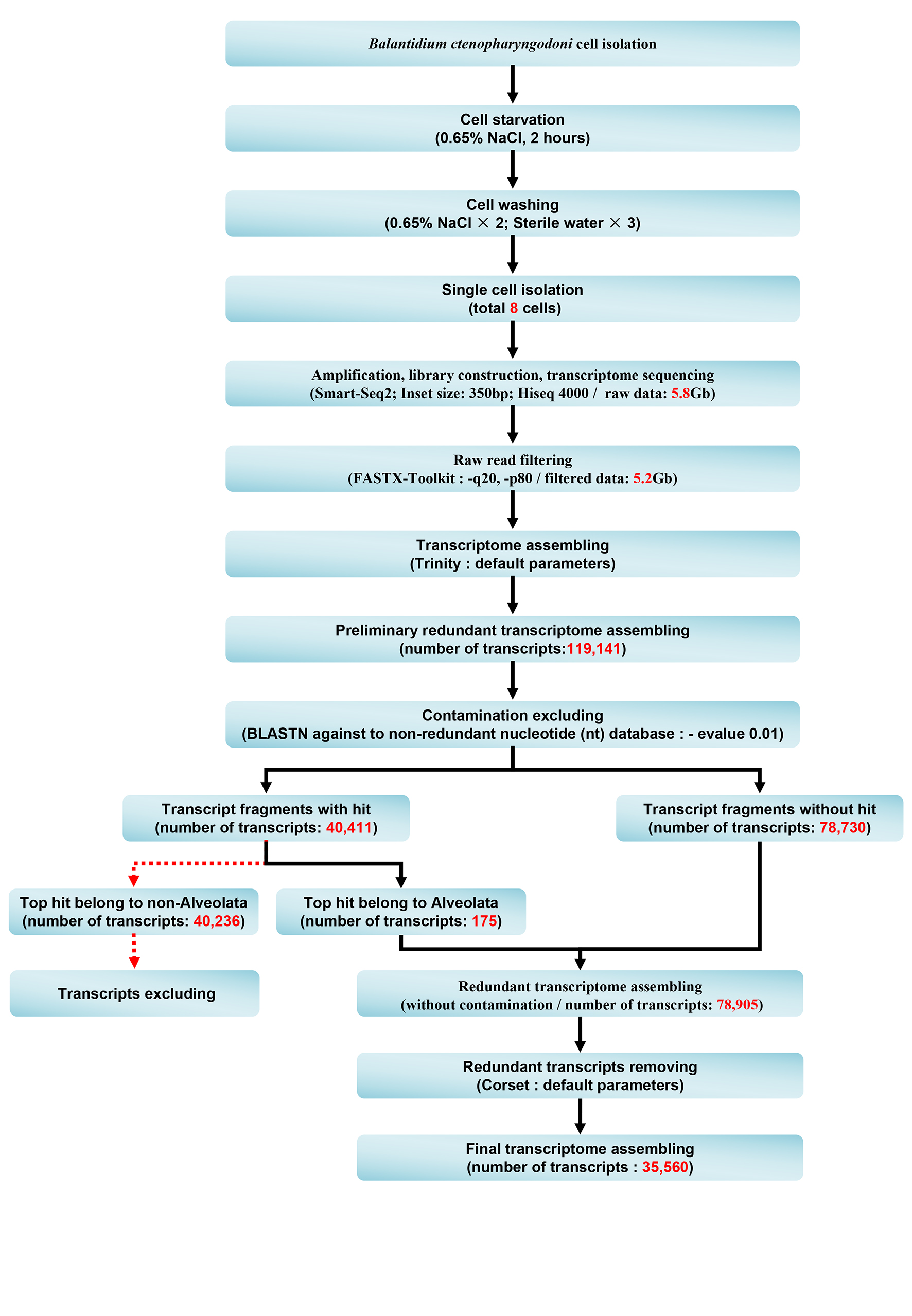

Supplement: Supplementary file 1 — Table S1 List of the omics data and SSU rDNA sequences used in the present study. Figure S1 The flowchart for obtaining transcriptome without contaminations and redundancy. Figure S2 Sequence alignments of SSU rDNA of B. ctenopharyngodoni. Black script, the length of fragment; blue script, the start site and termination site of alignment; red script, the identity of alignment; gray bar, two transcripts extracted from assembled transcriptome; green bar, GU48080 (B. ctenopharyngodoni small subunit ribosomal RNA gene, partial sequence); yellow bar, KU170972 (B. ctenopharyngodoni internal transcribed spacer 1, partial sequence; 5.8S ribosomal RNA gene, complete sequence; and internal transcribed spacer 2, partial sequence). Figure S3 Gene Ontology (GO) annotation of the final transcriptome. Figure S4 The guanine-cytosine (GC)-content distribution of B. ctenopharyngodoni's transcriptome. Yellow line, the GC content of preliminary redundant transcriptome; green line, the GC content of redundant transcriptome; red line, the GC content of final transcriptome. [file parasite-24-43-s1.zip › parasite170053-1-olm/Figure S1.jpg]

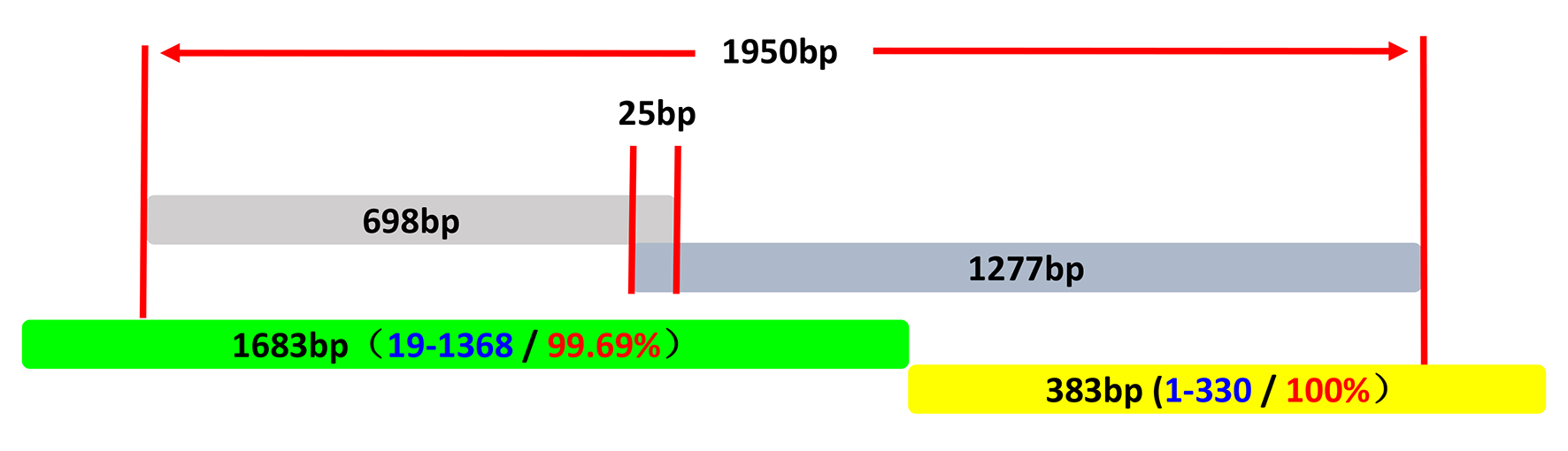

Supplement: Supplementary file 1 — Table S1 List of the omics data and SSU rDNA sequences used in the present study. Figure S1 The flowchart for obtaining transcriptome without contaminations and redundancy. Figure S2 Sequence alignments of SSU rDNA of B. ctenopharyngodoni. Black script, the length of fragment; blue script, the start site and termination site of alignment; red script, the identity of alignment; gray bar, two transcripts extracted from assembled transcriptome; green bar, GU48080 (B. ctenopharyngodoni small subunit ribosomal RNA gene, partial sequence); yellow bar, KU170972 (B. ctenopharyngodoni internal transcribed spacer 1, partial sequence; 5.8S ribosomal RNA gene, complete sequence; and internal transcribed spacer 2, partial sequence). Figure S3 Gene Ontology (GO) annotation of the final transcriptome. Figure S4 The guanine-cytosine (GC)-content distribution of B. ctenopharyngodoni's transcriptome. Yellow line, the GC content of preliminary redundant transcriptome; green line, the GC content of redundant transcriptome; red line, the GC content of final transcriptome. [file parasite-24-43-s1.zip › parasite170053-1-olm/Figure S2.jpg]

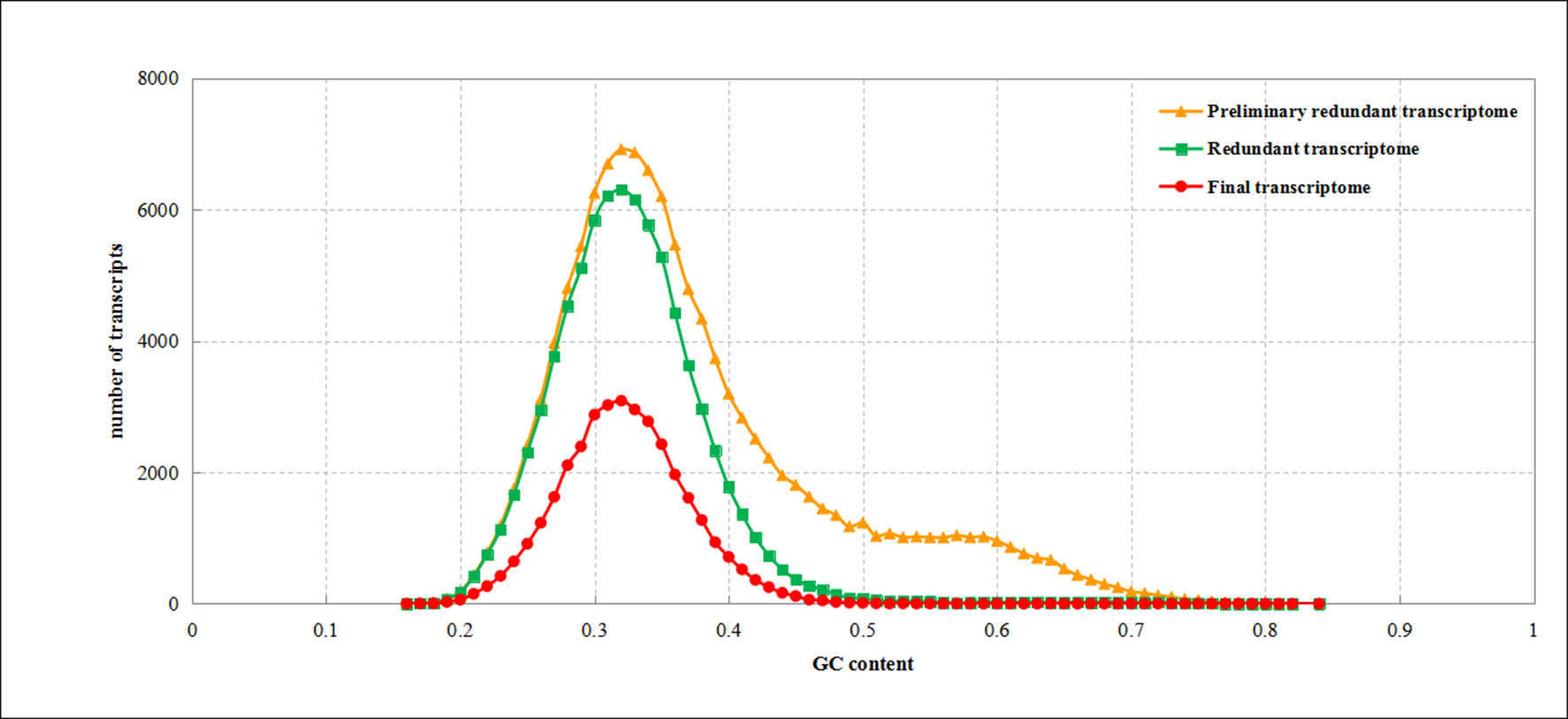

Supplement: Supplementary file 1 — Table S1 List of the omics data and SSU rDNA sequences used in the present study. Figure S1 The flowchart for obtaining transcriptome without contaminations and redundancy. Figure S2 Sequence alignments of SSU rDNA of B. ctenopharyngodoni. Black script, the length of fragment; blue script, the start site and termination site of alignment; red script, the identity of alignment; gray bar, two transcripts extracted from assembled transcriptome; green bar, GU48080 (B. ctenopharyngodoni small subunit ribosomal RNA gene, partial sequence); yellow bar, KU170972 (B. ctenopharyngodoni internal transcribed spacer 1, partial sequence; 5.8S ribosomal RNA gene, complete sequence; and internal transcribed spacer 2, partial sequence). Figure S3 Gene Ontology (GO) annotation of the final transcriptome. Figure S4 The guanine-cytosine (GC)-content distribution of B. ctenopharyngodoni's transcriptome. Yellow line, the GC content of preliminary redundant transcriptome; green line, the GC content of redundant transcriptome; red line, the GC content of final transcriptome. [file parasite-24-43-s1.zip › parasite170053-1-olm/Figure S4.jpg]
